# Methylglyoxal Evokes Pain by Stimulating TRPA1

**DOI:** 10.1371/journal.pone.0077986

**Published:** 2013-10-22

**Authors:** David A. Andersson, Clive Gentry, Emily Light, Nisha Vastani, Julie Vallortigara, Angelika Bierhaus, Thomas Fleming, Stuart Bevan

**Affiliations:** 1 Wolfson CARD, King’s College London, London, United Kingdom; 2 Department of Medicine I and Clinical Chemistry, University Hospital Heidelberg, Heidelberg, Germany; Indiana University School of Medicine, United States of America

## Abstract

Diabetic neuropathy is a severe complication of long-standing diabetes and one of the major etiologies of neuropathic pain. Diabetes is associated with an increased formation of reactive oxygen species and the electrophilic dicarbonyl compound methylglyoxal (MG). Here we show that MG stimulates heterologously expressed TRPA1 in CHO cells and natively expressed TRPA1 in MDCK cells and DRG neurons. MG evokes [Ca^2+^]_i_-responses in TRPA1 expressing DRG neurons but is without effect in neurons cultured from *Trpa1^−/−^* mice. Consistent with a direct, intracellular action, we show that methylglyoxal is significantly more potent as a TRPA1 agonist when applied to the intracellular face of excised membrane patches than to intact cells. Local intraplantar administration of MG evokes a pain response in *Trpa1^+/+^* but not in *Trpa1^−/−^* mice. Furthermore, persistently increased MG levels achieved by two weeks pharmacological inhibition of glyoxalase-1 (GLO-1), the rate-limiting enzyme responsible for detoxification of MG, evokes a progressive and marked thermal (cold and heat) and mechanical hypersensitivity in wildtype but not in *Trpa1^−/−^* mice. Our results thus demonstrate that TRPA1 is required both for the acute pain response evoked by topical MG and for the long-lasting pronociceptive effects associated with elevated MG *in vivo*. In contrast to our observations in DRG neurons, MG evokes indistinguishable [Ca^2+^]_i_-responses in pancreatic β-cells cultured from *Trpa1^+/+^* and *Trpa1^−/−^* mice. *In vivo,* the TRPA1 antagonist HC030031 impairs glucose clearance in the glucose tolerance test both in *Trpa1^+/+^* and *Trpa1^−/−^* mice, indicating a non-TRPA1 mediated effect and suggesting that results obtained with this compound should be interpreted with caution. Our results show that TRPA1 is the principal target for MG in sensory neurons but not in pancreatic β-cells and that activation of TRPA1 by MG produces a painful neuropathy with the behavioral hallmarks of diabetic neuropathy.

## Introduction

Sensory neuropathy is a common complication of type 1 and 2 diabetes and one of the major causes of neuropathic pain. The percentage of patients with neuropathy is generally considered to be about 50% for patients with long-standing diabetes and the prevalence is rising as a consequence of the increased global burden of type 2 diabetes [1]. The major symptom of diabetic polyneuropathy is sensory loss, but many patients suffer from a variety of abnormal sensations including paraesthesias, thermal and mechanical hypersensitivities, and spontaneous pain. Several processes have been suggested to contribute to the development of diabetic neuropathy. Prominent among these are oxidative stress and an increased formation of reactive carbonyl and dicarbonyl metabolites from glucose during hyperglycemia [2], [3]. We and others have shown that a number of lipid peroxidation products and reactive oxygen species (ROS) exert a direct pronociceptive action by stimulating an ion channel, TRPA1, expressed in nociceptive sensory neurons [4]–[8]. Although these reactive molecules do occur at increased levels during diabetes [9]–[12], increased production of these chemical factors is also seen in other pathologies that are not primarily characterized by sensory neuropathies (*e.g.* Alzheimer’s disease, Parkinson’s disease, amyotrophic lateral sclerosis and atherosclerosis) [13]. However, hyperglycemic episodes in diabetic patients are associated with an increased formation of the electrophilic reactive dicarbonyl compound methylglyoxal (MG). MG is primarily formed from triose intermediates in situations associated with increased glycolysis, such as hyperglycemia and fasting [14], [15]. During hyperglycemia, cells with an insulin independent glucose uptake (of particular importance in neurons) can experience dramatic increases in the rate of glycolysis [3]. Under these conditions, MG and other dicarbonyl containing glucose metabolites covalently modify proteins, giving rise to advanced glycation endproducts (AGE). The formation of AGE can directly interfere with the normal function of proteins, but also stimulates intracellular signaling pathways through activation of Receptor for AGE [16]. Here we confirm earlier reports that methylglyoxal (MG), one of the major dicarbonyl metabolites produced from glucose during hyperglycemia, stimulates the nociceptive ion channel TRPA1 [17], [18] directly through a reversible, intracellular interaction. *In vivo*, we show that pharmacologically elevated MG levels produce painful hypersensitivities to thermal and mechanical stimulation in wildtype, but not in *Trpa1^−/−^* mice. Our findings mirror clinical observations well, since altered sensitivity to thermal stimuli (hot and cold) produced by damage to small diameter nerve fibers is a reliable indicator that can distinguish between painful and painless diabetic neuropathy [19]. Together, these results demonstrate that increased concentrations of MG, in the absence of hyperglycemia, produce a sensory neuropathy similar to that seen in diabetic patients and in experimental animal models of diabetes.

## Experimental Procedures

### Cell Culture

DRG neurons were prepared from adult male or female mice using methods described previously [20]. Isolated neurons were maintained in MEM AQ (5.6 mM glucose) supplemented with 10% fetal bovine serum, 100 U/ml penicillin, 100 µg/ml streptomycin and 50 ng/ml NGF (Promega, Southampton, UK) for less than 24 hours before experimentation.

Mouse islets of Langerhans were isolated from the exocrine pancreas by collagenase digestion (1 mg/ml, type XI, Sigma, Poole, UK). Islets were dissociated by 15 min incubation in trypsin (0.5%, Sigma, Poole, UK) followed by trituration through a flame polished glass Pasteur pipette and the islet cells were seeded on glass cover slips.

MDCK cells were grown in DMEM (Invitrogen) supplemented with 5% fetal bovine serum, 100 U/ml penicillin and 100 µg/ml streptomycin.

Untransfected CHO cells and CHO cells expressing mouse or human TRPA1 were grown in MEM Glutamax or AQ medium supplemented with penicillin (100 U/ml), streptomycin (100 µg/ml), and FCS (10%). Plasmids encoding mutated TRPA1 channels were kindly provided by Dr. Ardem Patapoutian [21]. All media, serum and antibiotics were from Invitrogen (Paisley, UK) or Sigma (Poole, UK).

### Intracellular [Ca^2+^]_i_-measurements

Cells were loaded with 2 µM Fura-2 AM (Molecular Probes, Paisley, UK) in the presence of 1 mM probenecid for ∼1 hr. The experiments were performed in a physiological saline solution containing (in mM) 140 NaCl, 5 KCl, 10 glucose, 10 HEPES, 2 CaCl_2_, and 1 MgCl_2_, buffered to pH 7.4 with NaOH. Compounds were applied to cells by local continuous microperfusion of solution through a fine tube placed in close proximity of the cells studied. Images of a group of cells were captured every 2 sec using 340 and 380 nm excitation wavelengths with emission measured at 520 nm with a microscope based imaging system (PTI, New Jersey). Analyses of emission intensity ratios at 340 nm/380 nm excitation (R, in individual cells) were performed using the ImageMaster suite of software.

### 96 well plate assays

Changes in intracellular calcium ([Ca^2+^]_i_) in response to agonists were determined in TRPA1 expressing CHO cells and MDCK cells using a Flexstation 3 (Molecular Devices). Cells grown in 96 well black walled plates (Costar, Tewksbury, MA) were loaded with Fura 2-AM in the presence of 1 mM probenecid at 37°C for 1–1.5 hours and assays were carried out at 25°C. Basal emission ratios (340 nm/380 nm) were measured and changes in ratio determined at various times after compound addition.

### Electrophysiology

DRG neurons and TRPA1 CHO cells were studied under voltage-clamp conditions using an Axopatch 200B amplifier and pClamp 10.0 software (Molecular Devices, Sunnyvale, CA). Except where stated otherwise, whole cell recordings were performed at a holding potential of –60 mV using an extracellular solution with the composition described above for [Ca^2+^]_i_ measurements. Borosilicate glass pipettes (2–5 MΩ, 75–80% series resistance compensation) were filled with (in mM) 140 KCl, 1 CaCl_2_, 2 MgATP, 10 EGTA, and 10 HEPES buffered to pH 7.4 (KOH). Excised inside-out patches were recorded using a Ca^2+^-free solution containing (in mM) 140 NaCl, 5 KCl, 1 MgCl_2_, 10 HEPES and 1 EGTA, pH 7.4 (NaOH) in the pipettes and perfused with 140 KCl, 2 MgATP, 1 EGTA, and 10 HEPES (pH 7.4). DRG neurons were studied using an intracellular solution containing (in mM) 140 CsCl, 1 CaCl_2_, 2 MgATP, 10 EGTA and 10 HEPES, pH 7.4 (CsOH). Drugs were applied by local microperfusion with a rapid solution changer (RSC-200, Biologic, Claix, France).

### Ethics statement

All animal studies were carried out according to the U.K. Home Office Animal Procedures (1986) Act (project license PPL 70/0740) and were approved by the King’s College London Ethical Review Panel. Mice were killed by cervical dislocation.

### Behavioral experiments

Data shown are from male and female homozygote *Trpa1^−^* ^/*−*^ and *Trpa1* ^+/+^. The Trpa1-null mice and wild-type littermates were bred from heterozygotic mice provided by Drs. Kelvin Kwan and David Corey (Harvard Medical School, Boston, MA) [22].

Mechanical thresholds were measured using an Analgesymeter (Ugo-Basile, Milan). Mice were kept in their holding cages to acclimatize (10–15 min) to the experimental room. The experimenter then lightly restrained the mouse and applied an increasing pressure stimulus to the dorsal surface of the hind paw using a blunt conical probe. The nociceptive threshold was defined as the force in grams at which the mouse withdrew its paw. In order to avoid tissue injury a 150 g force cut-off value was used.

Tactile allodynia was assessed by measuring withdrawal thresholds to calibrated von Frey filaments (0.008–2 g). Animals were placed in a Perspex chamber with a metal grid floor giving access to the underside of their paws and allowed to acclimatize prior to the start of the experiment. Von Frey filaments were applied perpendicular to the mid plantar surface of the hind paw with sufficient force to cause slight bending against the paw and held for a few seconds. This was repeated 5 times at intervals of 1–2 s. A positive response was noted if the paw was sharply withdrawn or there was flinching upon removal of the hair. If no response was noted a higher force hair was tested. Using a modified ‘up-down’ method the lowest force filament producing a positive response was recorded as the threshold.

Heat and cold sensitivities were assessed by measuring the time for paw withdrawal from a 50°C hotplate or 10°C coldplate (Ugo Basile, Milan) of lightly restrained mice [23].

Methylglyoxal (250 nmoles in 25 µl saline) was injected subcutaneously into the plantar surface of one of the hind paws using a 50 µl luer-syringe (Hamilton Co.) fitted with a 26-gauge x 3/8 inch intradermal needle. Immediately after injection, mice were placed inside a Perspex chamber and the duration of the pain-related behaviors (licking and biting or flinching and shaking of the injected paw) recorded using a digital stop-watch. Total pain response times over the first 2 minutes were used for analysis as the pain behaviors were largely confined to this period. The glyoxalase 1 inhibitor Sr-p-Bromobenzylglutathione cyclopentyl diester (50 mg/kg) was dissolved in 2% DMSO/20% Tween 80 in saline and administered by intraperitoneal (i.p.) injection (5 ml/kg).

The glucose tolerance test was performed in mice (not fasted) treated i.p. with 2 g/kg glucose or vehicle (0.2 ml, 0.9% saline). HC030031 (100 mg/kg) or vehicle (DMSO 10%, Tween 80 5%, 0.9% saline 85%) was administered i.p. 30 min before glucose or vehicle. Glucose concentrations were determined using a Contour blood glucose meter (Bayer Diabetes Care) in blood samples taken from the tail-tip.

### Immunohistochemistry

Formalin fixed plantar skin was embedded in paraffin wax and cut in 8 µm sections and stained with rabbit polyclonal anti-PGP9.5 (1 in 5000, Ultraclone Ltd, UK) after citric acid heat induced epitope retrieval. Staining was visualized using anti-rabbit IgG labeled with Alexa-594. The number of PGP9.5 positive fibers crossing the basal membrane into the epidermis was determined in n = 6 mice for each group on a Zeiss Axioplan 2 microscope. The observer was blinded to the treatment.

### Drugs and chemicals

The glyoxalase 1 inhibitor Sr-p-Bromobenzylglutathione cyclopentyl diester was provided by Dr. Thomas Fleming and was prepared and characterized as previously described [24], [25]. AP18, 4-(4-chlorophenyl)-3-methylbut-3-en-2-one oxime, was from Maybridge (Tintagel, UK). Methylglyoxal and all other reagents were from Sigma (Poole, UK).

### Statistical analysis

Data are presented as mean ± s.e.m and the number of experimental observations (animals or cells) is indicated by n. Data were analyzed by t-test or ANOVA followed by Dunnett’s or Tukey’s post-hoc test for multiple comparisons.

## Results

### Methylglyoxal stimulates heterologously expressed TRPA1

**Figure 1:**
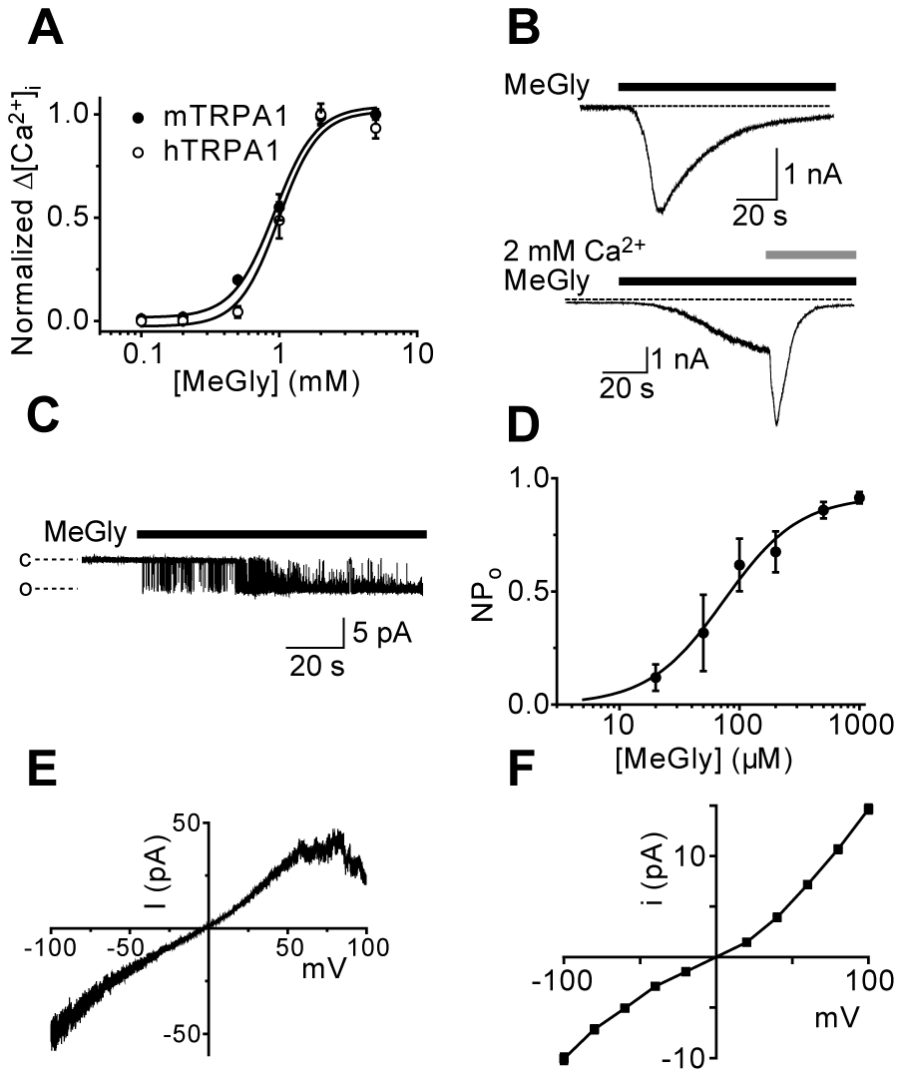
Methylglyoxal is an intracellular TRPA1 agonist. (A) Application of MG evokes concentration-dependent [Ca^2+^]_i_-responses in CHO cells expressing mouse or human TRPA1. (B) MG (0.5 mM) elicits inward currents with the characteristic rapid onset and inactivation in the presence of 2 mM Ca^2+^ (top panel). In Ca^2+^-free solutions (1 mM EGTA) the inward current grew more slowly and addition of Ca^2+^ produced the typical current surge followed by a rapid inactivation of the current (bottom panel). (C) MG rapidly activates TRPA1 in excised inside-out patches, with a markedly higher potency than when applied extracellularly (D, compare with A). (E) The current voltage relationship in membrane patches containing several channels demonstrated a reversal potential close to 0 mV and channel block or inactivation at positive membrane potential. (F) Current-voltage relationship for a single channel in an inside-out patch stimulated with MG.

We examined the effect of MG on [Ca^2+^]_i_ in untransfected CHO cells and CHO cells expressing TRPA1. MG evoked concentration dependent increases in [Ca^2+^]_i_ in cells expressing mouse TRPA1 (EC_50_ = 0.9±0.1 mM) or human TRPA1 (EC_50_ = 0.7±0.2 mM; Fig. 1A), but had no effect on [Ca^2+^]_i_ in untransfected CHO cells or in the absence of extracellular Ca^2+^ (data not shown), demonstrating that MG evokes [Ca^2+^]_i_-responses by stimulating Ca^2+^-influx through TRPA1. In whole cell voltage-clamp experiments, MG produced inward currents that displayed a current waveform with Ca^2+^-mediated potentiation and inactivation similar to that previously observed with other TRPA1 agonists (Fig. 1B; [4], [26]). These results agree well with those of earlier reports characterizing MG as a TRPA1 agonist [17], [18], [27]. Since methylglyoxal is a reactive, short-lived molecule formed intracellularly [28], [29] and the TRPA1 residues targeted by electrophilic agonists are located intracellularly [30], [31], we examined the effect of MG applied to the intracellular face of excised inside-out patches (Fig. 1CD). In this configuration, MG rapidly stimulated TRPA1 channel activity and was markedly more potent than when applied extracellularly (see Fig. 1A). MG stimulated TRPA1 channel activity with an EC_50_ of 74 µM in the inside-out configuration (Fig. 1D), strongly suggesting that methylglyoxal acts at an intracellular site. TRPA1 channels activated by methylglyoxal in excised inside-out patches displayed a voltage dependent inactivation or block at positive membrane potentials (Fig. 1E), similar to that observed with other TRPA1 agonists [4]. The single channel conductance was larger at positive membrane potentials (120 pS at +60 mV and 150 pS at +100 mV) and somewhat smaller (70-80 pS) in the physiological voltage range (–20 to –60 mV; Fig. 1F), consistent with the properties of TRPA1 [32].

### Methylglyoxal stimulates TRPA1 in MDCK cells

**Figure 2:**
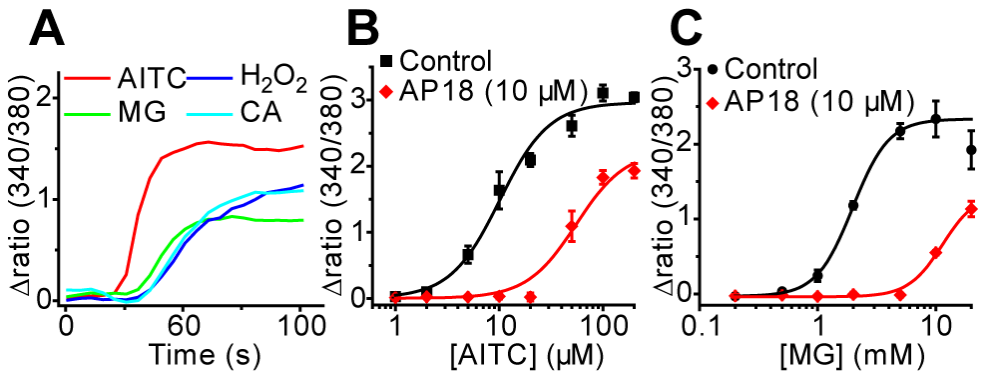
TRPA1 is expressed in MDCK cells. (A) The TRPA1 agonists AITC, H_2_O_2_, MG and cinnamaldehyde stimulate [Ca^2+^]_i_-responses in MDCK cells. (B, C) The selective TRPA1 antagonist AP18 (10 µM) produces a rightward shift of AITC and MG evoked [Ca^2+^]_i_-responses in MDCK cells.

MG has previously been reported to stimulate [Ca^2+^]_i_-influx through an unknown mechanism in Madin-Darby Canine Kidney (MDCK) cells [33]. We used [Ca^2+^]_i_-measurements to determine the involvement of TRPA1 in MG evoked [Ca^2+^]_i_-responses in MDCK cells. As illustrated in Fig. 2A, MG (5 mM) and the TRPA1 agonists AITC (100 µM), cinnamaldehyde (1 mM) and hydrogen peroxide (20 mM) all evoked [Ca^2+^]_i_-influx responses in MDCK cells (Fig. 2A). In concentration-response experiments, MG evoked [Ca^2+^]_i_-responses with an EC_50_ of 2.2 ± 0.4 mM and AITC produced an EC_50_ value of 9.1 ± 2.0 µM (Fig. 2BC). AP18 is a selective and potent inhibitor of TRPA1 [34], which likely acts through a saturable allosteric rather than a competitive mechanism [35]. AP18 (10 µM) produced similar rightward shifts of the concentration-response curves for AITC and MG in MDCK cells, indicating a common target in TRPA1 (Fig. 2BC).

### TRPA1 mediates methylglyoxal responses in DRG neurons

**Figure 3:**
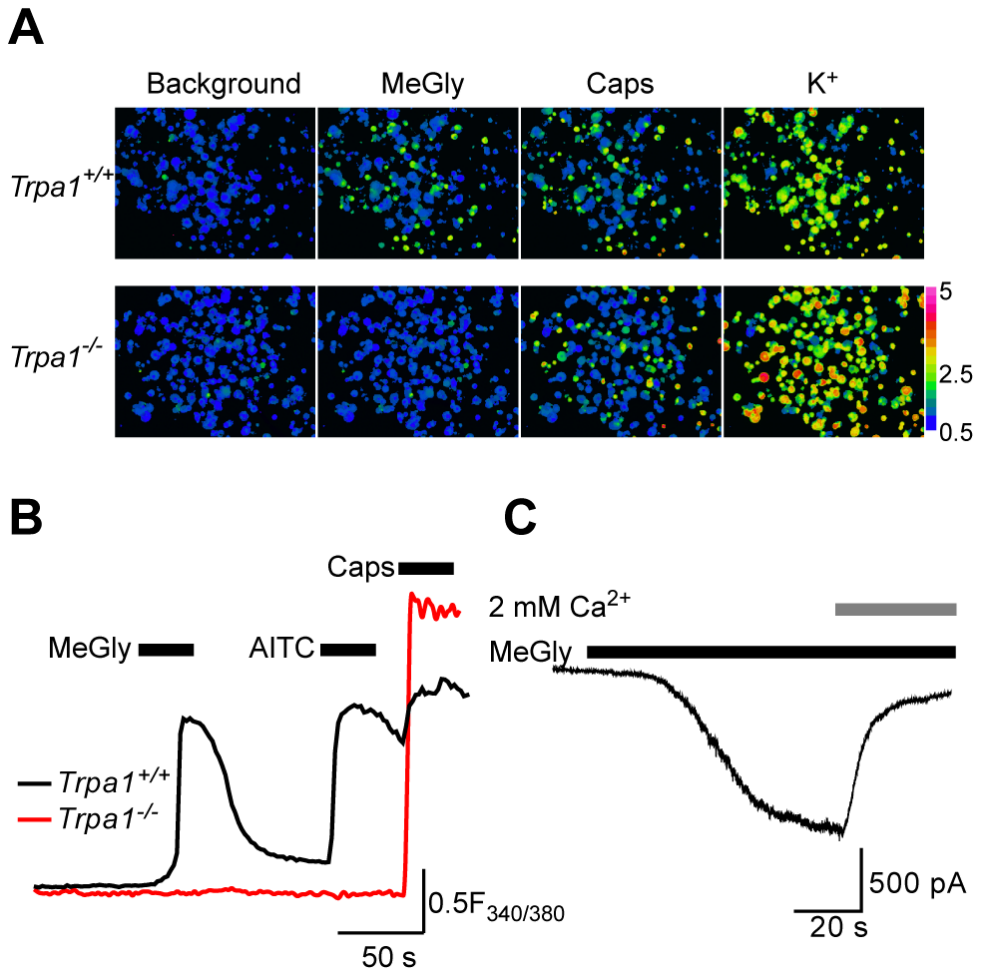
Methylglyoxal stimulates DRG neurons expressing TRPA1. (A) Pseudocoloured images illustrating [Ca^2+^]_i_-concentrations in Fura-2 loaded cultured DRG neurons. MG evokes [Ca^2+^]_i_-responses in TRPA1 containing neurons. (B) Typical examples of [Ca^2+^]_i_-responses in capsaicin sensitive DRG neurons from *Trpa1^+/+^* and *Trpa1^−/−^* mice. (C) MG activates characteristic TRPA1 currents in DRG neurons (holding potential -60 mV). Before addition of 2 mM Ca^2+^ this neuron was superfused with a solution containing 15 µM Ca^2+^, which prevents the Ca^2+^-induced current surge.

TRPA1 is a sensory neuron transduction channel for pronociceptive irritant chemicals and inflammatory mediators [22], [36] and we next determined whether MG stimulates nociceptive neurons directly. Similar to the results described by Eberhardt et al., (2012), we found that MG induced [Ca^2+^]_i_-responses in 72% (240 of 333) of capsaicin-sensitive (TRPV1 positive) DRG neurons cultured from *Trpa1^+/+^* mice, but failed to increase [Ca^2+^]_i_ in DRG neurons (0%, 1 of 322) cultured from *Trpa1^−/−^* mice (Fig. 3AB). We also examined the effect of MG on DRG neurons using voltage clamp recordings (Fig. 3C). In a proportion of DRG neurons MG evoked robust inward currents (in solutions containing 15 µM Ca^2+^ which prevents the Ca^2+^ mediated current surge [4], [37]) that were inactivated by readmission of 2 mM Ca^2+^ in the same way as currents evoked by other electrophilic TRPA1 agonists in DRG neurons [4], [26].

### Site of action

**Figure 4:**
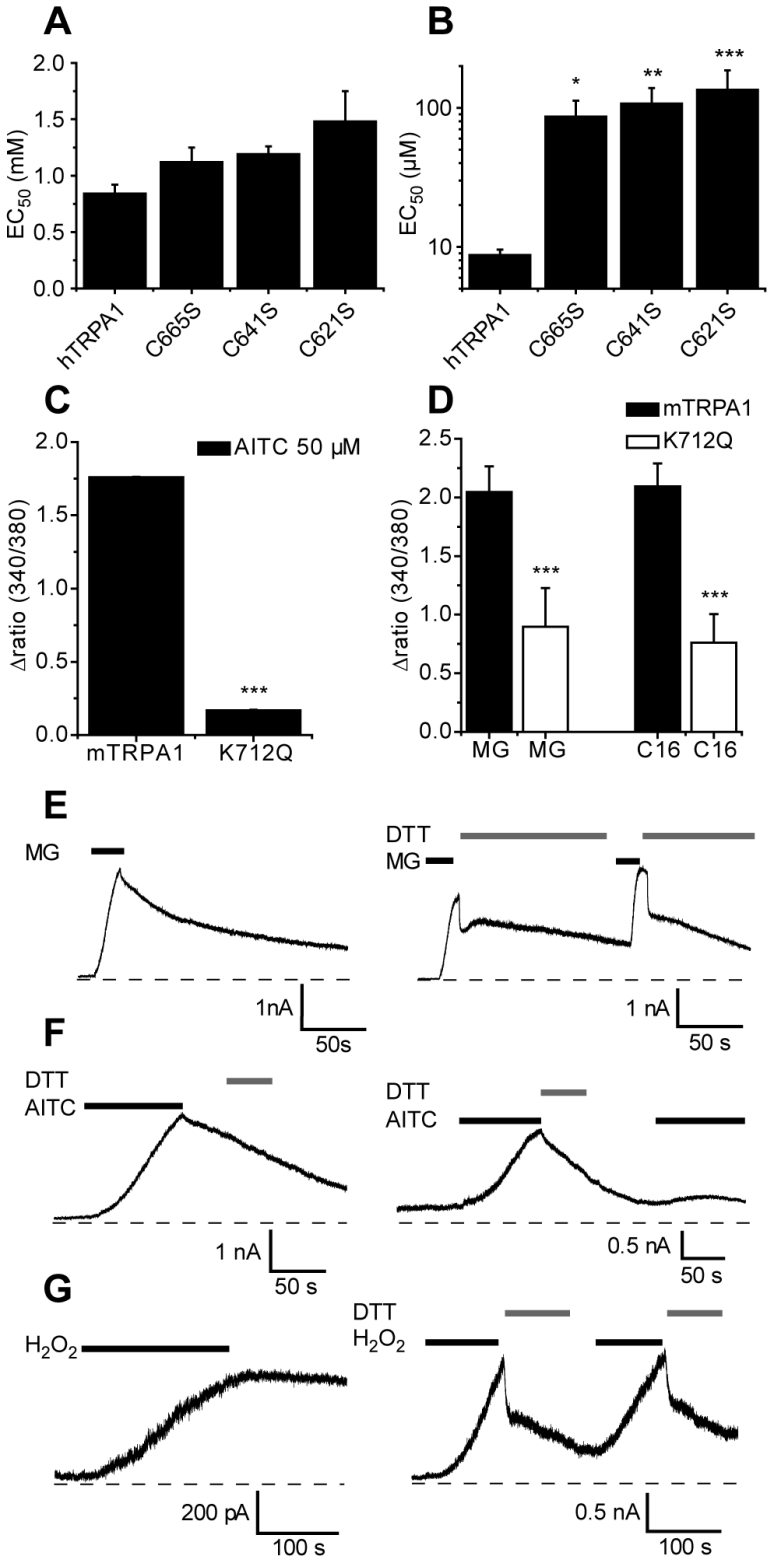
Methylglyoxal is a reversible TRPA1 agonist. The cysteine substitutions C665S, C641S and C621S (in human TRPA1) do not significantly affect responses evoked by MG (A), but dramatically reduce the sensitivity to AITC (B). (C) The lysine substitution K712Q (mouse TRPA1) reduced [Ca^2+^]_i_-responses evoked by AITC (50 µM). (D) The K712Q mutation reduced the amplitude of [Ca^2+^]_i_-responses evoked by MG (2 mM) and Δ^9^-tetrahydrocannabiorcol (20 µM; C16) in individual CHO cells (n = 22–64). (E) Outward TRPA1 current responses (+60 mV) evoked by MG (1 mM) in CHO cells decay relatively slowly when MG is removed (recorded under Ca^2+^-free conditions). Application of DTT (2 mM) produces a rapid, partial current inactivation. (F) Currents evoked by AITC (50 µM) under the same conditions inactivate relatively slowly, but are resistant to DTT and leave TRPA1 refractory to stimulation. (G) Currents elicited by the oxidant H_2_O_2_ (5 mM) remain at a stable level after removal of H_2_O_2_, but are rapidly and reversibly inactivated by DTT. Data were analyzed by ANOVA followed by Dunnett’s *post-hoc* test (panel B) or by t-test (*P<0.05, **P<0.01, ***P<0.001 compared to the wildtype channel).

Electrophilic TRPA1 agonists stimulate the channel through covalent modification of N-terminal cysteine residues [30], [31]. To determine whether the TRPA1 cysteine residues that have previously been identified as targets for covalent modification by electrophilic agonists influence the sensitivity to MG, we examined the effect of MG on human TRPA1 channels containing the single point mutations C621S, C641S or C665S (Fig. 4A). None of these amino acid substitutions affected the potency of MG significantly, but each substitution very substantially reduced the TRPA1 sensitivity to AITC (Fig. 4B). Although many of the 31 cysteine residues present in TRPA1 can be modified by electrophilic agents [31], a limited number of these residues are required for channel activation [30], [31]. Furthermore, it is likely that the relative importance of different residues varies between agonists [38].

In addition to the N-terminal cysteine residues, lysine K712 (in mouse) has been implicated as a target for electrophilic TRPA1 agonists [30]. We therefore examined the effect of the point mutation K712Q on agonist responses evoked by MG, AITC and the non-covalent agonist Δ^9^-tetrahydrocannabiorcol [39]. The amplitude of [Ca^2+^]_i_-responses evoked by AITC (50 µM), MG (2 mM) and Δ^9^-tetrahydrocannabiorcol in cells expressing mTRPA1 K712Q were all significantly reduced compared to those in WT mTRPA1 (Fig. 4CD). The fact that the sensitivity to all three agonists was reduced in the K712Q mutant indicates that this residue is important for the functional integrity of TRPA1 and does not exclusively act as a nucleophilic acceptor for electrophilic compounds.

It is possible that MG stimulates TRPA1 through formation of reversible hemithioacetals with cysteine residues other than those examined here [18], [40]. To investigate this possibility we examined the effect of the reducing agent dithiothreitol (DTT, 2 mM) on outward TRPA1 currents recorded in the absence of Ca^2+^ (5 mM BAPTA intracellularly, 1 mM EGTA extracellularly) to avoid Ca^2+^-mediated inactivation or desensitization. We used DTT to determine whether disulfide bridge formation or reversible noncovalent interactions contribute to the agonist activity of MG. DTT is expected to rapidly reduce cystine bridges to free thiols, but also to form hemithioacetals with MG, thereby accelerating current decay. Covalent modification of arginine and lysine residues by MG and modification of cysteine residues by AITC are on the other hand resistant to DTT [31]. Under these recording conditions, MG rapidly evoked an outward TRPA1 current that partially decayed with time after removal of MG (Fig. 4E), suggesting a reversible interaction between MG and TRPA1. Application of DTT immediately after MG removal dramatically accelerated the TRPA1 current decay consistent with a reversible interaction (Fig. 4E). When current responses evoked by a pharmacologically active concentration of H_2_O_2_ were examined in the same way, it was notable that they remained at a stable amplitude after removal of H_2_O_2_, but that application of DTT rapidly reduced the current amplitude (Fig. 4G). This is consistent with the expected oxidative formation of cystine bridges and their reduction by DTT [4]. The contrast between the stable currents produced by H_2_O_2_ and the spontaneously reversible currents evoked by MG highlights the reversible nature of the interaction between MG and TRPA1. Currents evoked by AITC declined with time after removal of AITC, but were resistant to treatment with DTT (Fig. 4F). We also observed that repeated applications of MG and H_2_O_2_ evoked reproducible current responses of similar amplitudes, whereas an application of AITC prevented subsequent channel activation by AITC challenges (Fig. 4C-E). We conclude that MG acts through a reversible interaction with TRPA1 and that H_2_O_2_ relies on DTT sensitive oxidative modification of TRPA1 protein for its activity. These results strongly suggest that MG primarily acts through hemithioacetal formation as concluded by Eberhardt *et al.* [18]. In contrast, the covalent modification produced by AITC appears irreversible and renders TRPA1 refractory to subsequent activation.

### MG evokes pain and neuropathy by stimulating TRPA1

**Figure 5:**
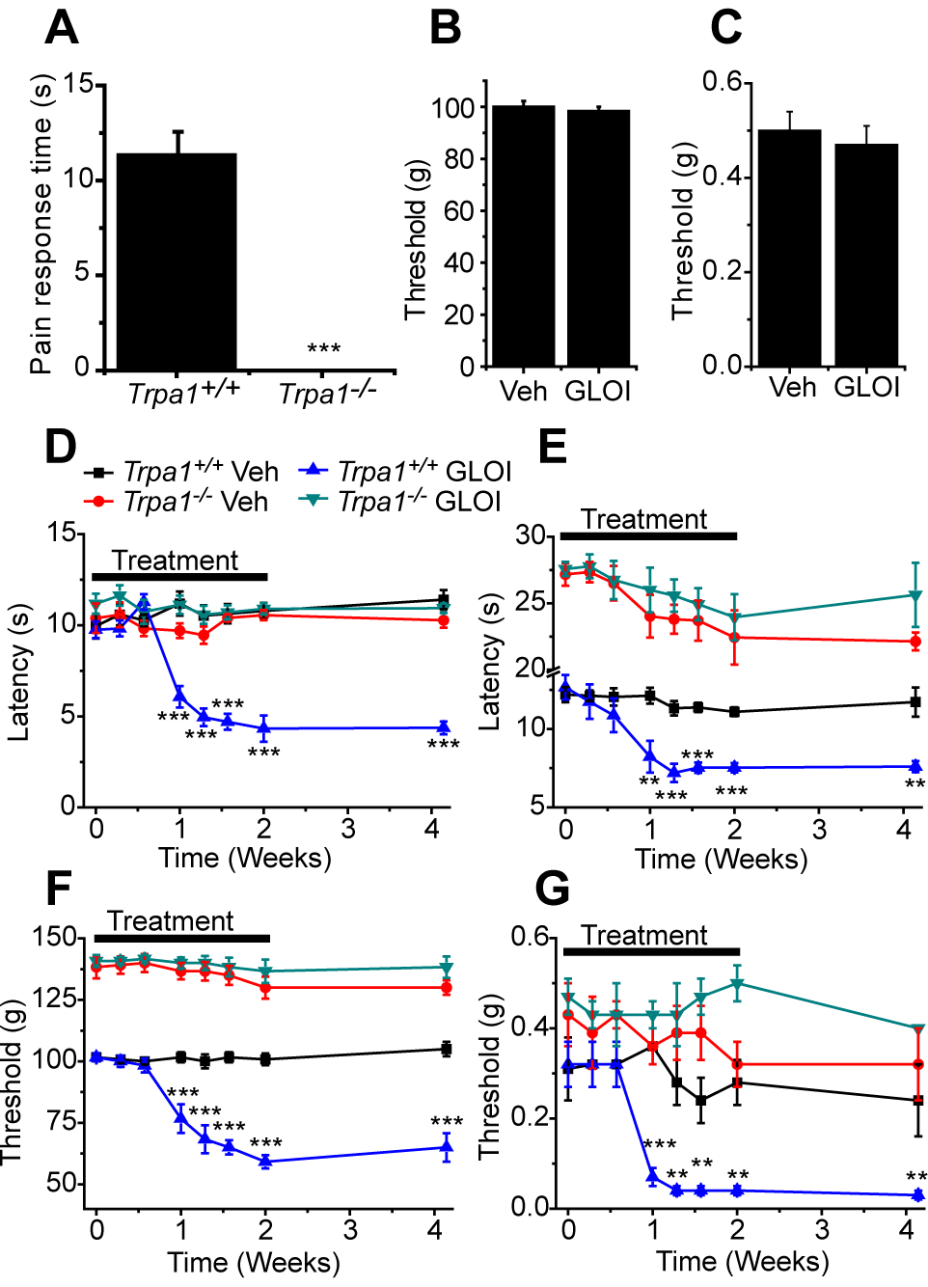
Methylglyoxal produce persistent sensory neuropathy. (A) Intraplantar injections of MG (250 nmoles in 25 µl) evokes nociceptive behaviors in wildtype mice but is without effect in *Trpa1^−/−^* mice (n = 6). Injections of the GLO-1 inhibitor Sr-p-Bromobenzylglutathione cyclopentyl diester (GLOI, 50 mg/kg, 1h before test) did not acutely affect the withdrawal threshold in the paw pressure (B) or von Frey (C) tests compared to mice treated with vehicle (n = 6). (D-G) Effect of GLOI or vehicle injections administered every 2 days during a 2 week treatment period on the paw withdrawal latency from a 50°C hotplate (D), 10°C coldplate (E), the paw withdrawal threshold in the paw pressure test (F) and in response to stimulation with von Frey filaments (G). The data points are mean ± SEM from n = 6 mice, except for the last time point (15 days after the final injection) where n = 3. Data were analyzed by t-test (panel A) or ANOVA followed by Tukey’s HSD test (**P<0.01, ***P<0.001 compared to vehicle treated group).

Local administration of TRPA1 agonists evokes acute pain [41]–[44] and we therefore examined whether this is also the case with MG. *Trpa1^+/+^* and *Trpa1^−/−^* mice were given intraplantar injections of MG (250 nmoles/paw) and the time spent dedicated to pain-related behaviors during the 2 min following injection was quantified. MG induced a significant nociceptive response in *Trpa1^+/+^* mice, but was without effect in *Trpa1^−/−^* mice, confirming that TRPA1 is required for the pronociceptive effect of MG *in vivo*, even after administration of a relatively high bolus dose (Fig. 5A).

Although acute administration of MG identified a potential role for TRPA1 as a sensor of elevated MG levels, it is not an experimental situation that accurately reflects the long-lasting, widely increased MG levels associated with hyperglycemia in diabetes. To model this situation more closely, we made use of the selective glyoxalase-1 (GLO-1) inhibitor Sr-p-Bromobenzylglutathione cyclopentyl diester [45]. GLO-1 is the rate-limiting enzyme responsible for the hydrolysis of the majority of MG formed *in vivo* and inhibition of this enzyme significantly increases MG concentration in cultured neuronal and non-neuronal cells, as well as in plasma and brain *in vivo* [45], [46]. Acute inhibition of GLO-1 did not influence nociception, since the sensitivities in the paw pressure and von Frey filaments tests were unaffected 1 h after administration of the GLO-1 inhibitor (Fig. 5B,C).

To determine the effect of prolonged elevation of MG levels *in vivo* on nociception, we administered the GLO-1 inhibitor on 7 occasions over 2 weeks to *Trpa1^+/+^* and *Trpa1^−/−^* mice [47]. During the course of the experiment we examined the nociceptive responses to cold, heat and mechanical stimulation (using the paw-pressure test and punctate stimulation with von Frey filaments) on days following administration of the GLO-1 inhibitor. Consistent with the expected time-course of the pharmacologically induced increase in MG concentrations [47], we observed no increased sensitivity in any of the tests during the first 4 days of the study (Fig. 5D-G). This indicates that the GLO-1 inhibitor itself is well tolerated and is without any direct effect on nociception. After day 4, the sensitivity to all four test modalities was progressively increased in the *Trpa1^+/+^*, but not the *Trpa1^−/−^* mice, reaching a plateau after 1–2 weeks of dosing. The observed hypersensitivities were long-lasting, with no sign of reversal even two weeks after cessation of drug administration.

### Inhibition of GLO-1 does not reduce the number of intraepidermal nerve fibers

**Figure 6:**
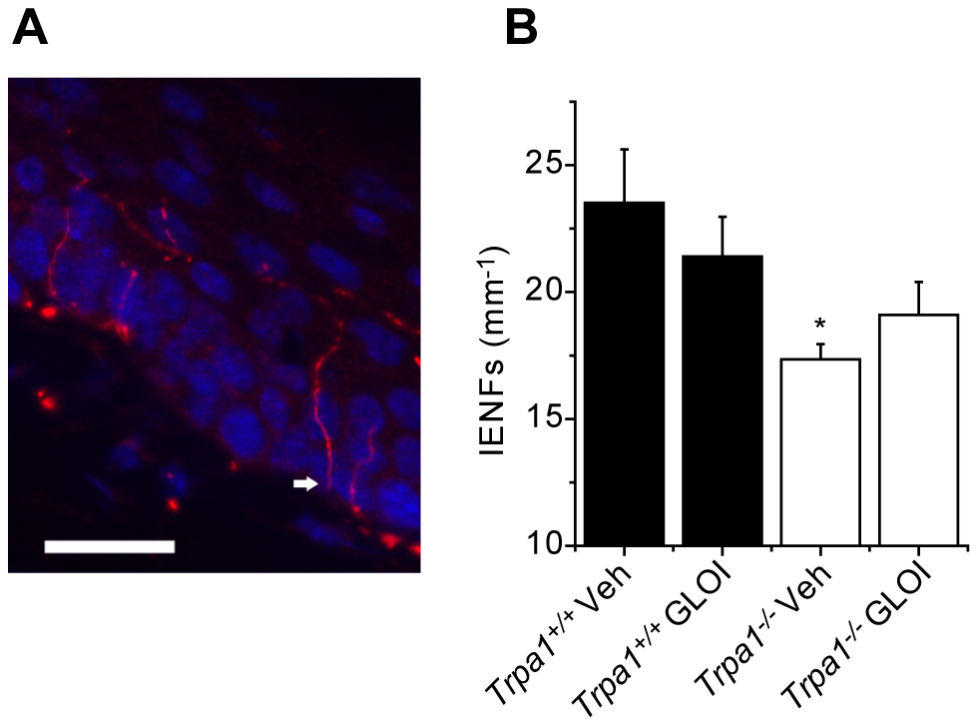
Intra-epidermal nerve fiber density is unaffected by 2 weeks GLO-1 inhibition. (A) PGP9.5 positive nerve fibers crossing the basal membrane into the epidermis (the white arrow highlights an example) in 8 µm sections of plantar skin (scale bar = 20 µm). (B) Following two weeks treatment with the GLO-1 inhibitor (GLO-I, 50 mg/kg every other day), the number of intra-epidermal nerve fibers crossing into the epidermis/mm skin was unchanged in both *Trpa1^+/+^* mice and in *Trpa1^−/−^* littermates. Data were analyzed by ANOVA followed by Tukey’s HSD test (n = 6 mice, *P<0.05 compared to vehicle treated *Trpa1^+/+^* mice).

The early stages of diabetic neuropathy are characterized by damage to small nerve fibers and a reduction of intraepidermal nerve fibers (IENFs) is a useful clinical diagnostic tool for sensory neuropathies of different etiologies [48], [49]. We examined skin sections from *Trpa1^+/+^* and *Trpa1^−/−^* mice treated with the GLO-1 inhibitor or vehicle for 2 weeks to assess whether the hypersensitivities produced by inhibition of GLO-1 were accompanied by a loss of IENFs, similar to that seen in mouse models of diabetes. Inhibition of GLO-1 failed to produce a significant reduction of the IENF density compared to that seen in vehicle treated mice in either genotype (Fig. 6AB), suggesting that 2 weeks of increased MG levels is not sufficient to damage IENFs. The density of IENFs was significantly lower in vehicle treated *Trpa1^−/−^* mice compared to wildtype littermates (Fig. 6B). It is possible that this anatomical difference between the two genotypes contributes to the reduced sensitivity to cold and mechanical stimuli in naïve TRPA1-null mice [22], [41].

### MG stimulates mouse pancreatic beta cells independently of TRPA1

**Figure 7:**
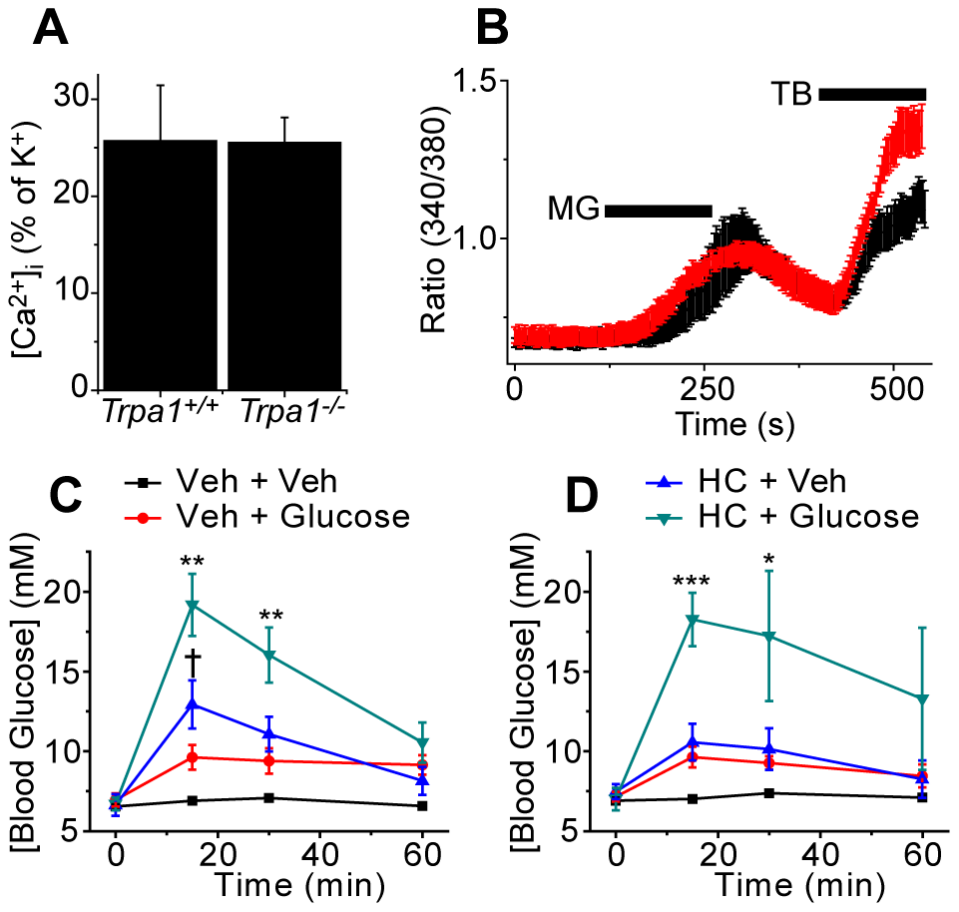
TRPA1 does not influence insulin release. (A) MG (0.5 mM) evokes indistinguishable [Ca^2+^]_i_-responses in β-cells from *Trpa1^+/+^* (black) and *Trpa1^−/−^* (red) mice (n = 15-28). (B) Illustration of the time course of MG and tolbutamide (200 µM) evoked [Ca^2+^]_i_-responses in β-cells. (C, D) Effect of the TRPA1 antagonist HC030031 (HC, 100 mg/kg) or vehicle (Veh) administered 30 min before glucose or vehicle in the glucose tolerance test (2 g/kg, *i.p.*) performed in *Trpa1^+/+^* (C) and *Trpa1^−/−^* mice (D). Data points are mean ± s.e.m of n = 6 mice and were analyzed by ANOVA followed by Tukey’s HSD test (*P<0.05, **P<0.01, ***P<0.001 compared to the group treated with vehicle and glucose, †P<0.05 compared to the group treated with vehicle and vehicle).

‘MG formed from glucose has been suggested to stimulate basal insulin release in response to elevated glucose concentrations through activation of TRPA1 expressed in pancreatic β-cells [17]. To identify a possible role for TRPA1 as a MG receptor in beta cells directly, we compared the effect of MG and AITC on beta cells cultured from *Trpa1^+/+^* and *Trpa1^−/−^* mice.. AITC failed to elicit [Ca^2+^]_i_-responses (data not shown) in beta cells cultured from either wildtype or *Trpa1^−/−^* mice, suggesting that mouse beta cells do not express TRPA1. Intriguingly, MG evoked [Ca^2+^]_i_-responses in a subpopulation of tolbutamide sensitive beta cells, but the number of MG sensitive cells and the response amplitude evoked by MG were similar in cultures from *Trpa1^+/+^* and *Trpa1^−/−^* mice, demonstrating that MG stimulates beta cells independently of TRPA1, at least in the mouse (Fig. 7AB).

We also examined whether TRPA1 influences blood glucose levels *in vivo*. In this experiment we examined the effect of the TRPA1 antagonist HC030031 (100 mg/kg, i.p.) on the elevation in blood glucose evoked by administration of glucose (2 g/kg in 0.2 ml, i.p.). Administration of glucose produced similar increases in blood glucose concentration in mice of both genotypes (Fig. 7CD). A notable finding was that administration of HC030031 dramatically enhanced the increase in blood glucose seen following a glucose challenge, both in *Trpa1^+/+^* and in *Trpa1^−/−^*, suggesting that this antagonist interferes with glucose metabolism independently of TRPA1. Moreover, HC030031 also increased the blood glucose in mice that were not challenged with glucose (Fig. 7CD).

## Discussion

We have demonstrated that MG stimulates natively expressed TRPA1 in DRG neurons and MDCK cells and that acute administration of MG evokes pain mediated by TRPA1. We exploited a pharmacological inhibitor of GLO-1 to raise the levels of MG for a period of 2 weeks [47] and observed a progressive development of hypersensitivities to cold, heat and mechanical stimuli. Critically, we found that TRPA1 is required for the development of painful hypersensitivities and our results thus demonstrate that persistent stimulation of TRPA1 is sufficient to produce some of the well-established sensory characteristics of painful diabetic sensory neuropathy.

Glucose uptake is insulin-independent in sensory neurons and can therefore vary dramatically with fluctuations in plasma glucose [3]. Increased glucose flux during hyperglycemia in turn leads to a dramatic increase in the intracellular formation of MG [14], [47]. Increased formation of MG during hyperglycemia in diabetic patients is considered a major causal factor for the development of diabetic sensory neuropathy and other long-term diabetic complications [14], [15], [47], [50]. It has recently been proposed that MG elicits sensory neuron hypersensitivity by modifying the properties of a sub-type of voltage gated sodium channel, Na_V_1.8, [47]. Our results demonstrate that a direct effect of MG on Na_V_1.8 is not sufficient to produce pain or hypersensitivity in the absence of TRPA1, but suggest that actions at both TRPA1 and NaV1.8 are required for the behavioral sensitization observed following treatment with a GLO-1 inhibitor.

The chemotherapeutic agents paclitaxel and oxaliplatin produce persistent, TRPA1-dependent pain and hypersensitivities [51], [52], similar to our results with GLO-1 inhibition. Together, these findings are consistent with the notion that TRPA1 is central for the development of several common and clinically important classes of painful metabolic or pharmacologically induced neuropathies.

### MG as a TRPA1 agonist

MG evokes [Ca^2+^]_i_-responses in cultured DRG neurons, MDCK cells and TRPA1 expressing CHO cells by stimulating TRPA1. Studies of MG modification of proteins have shown that MG forms reversible hemithioacetals with cysteine residues but stable covalent glycation products with lysine and in particular, arginine residues [40]. Our results are consistent with a reversible interaction between MG and cysteines, since application of DTT largely reversed TRPA1 currents evoked by MG [53]. This conclusion is further supported by the fact that application of MG to the intracellular face of excised patches stimulates TRPA1 with an apparent affinity that is close to estimates for the interaction between MG and N-acetylcysteine [40]. Our observation that the amino acid substitutions C621S, C641S, and C665S did not affect the potency of MG whereas each mutation substantially reduced the TRPA1 sensitivity to AITC, is consistent with MG stimulating TRPA1 through formation of hemithioacetals [54], a mechanism distinct from that underlying the agonist effect of AITC [30]. It is possible that cysteine residues other than those examined here are targeted by MG and the possibility that unrelated cysteines can compensate for the loss of the mutated cysteines involved in MG induced disulfide formation cannot be excluded [18]. In agreement with the results presented here, C641 has been identified as a critical residue for the agonist activity of AITC [30], but this residue has also been shown not to influence the sensitivity of TRPA1 to cinnamaldehyde [21]. Taken together, these observations therefore suggest that different electrophilic cysteine-reactive TRPA1 agonists do not interact with an identical subset of TRPA1 cysteine residues [38]. The small DTT resistant component of the MG evoked current may involve covalent modification of arginine or lysine residues [55]. Lysine 712 (K710 in human TRPA1) has previously been identified as a target for AITC, but in our hands appeared to compromise normal channel function, since it reduced TRPA1 sensitivity to MG, AITC and the non-covalently acting agonist Δ^9^-tetrahydrocannabiorcol to a similar extent.

TRPA1 is expressed in the gastrointestinal tract [56], but to our knowledge this is the first demonstration of functional TRPA1 channels in a cell line derived from the kidney. Expression of TRPA1 in MDCK cells suggests that some agents that have previously been shown to increase [Ca^2+^]_i_ in MDCK cells, may do so by stimulating TRPA1 [57], [58].

MG has been proposed to regulate insulin release *in vivo* and in rat pancreatic β-cells by stimulating TRPA1 [17]. Our results demonstrate that this is not the case in the mouse. Although MG evoked [Ca^2+^]_i_-responses in mouse β-cells, these were identical in cultures from wildtype and TRPA1-null mice and thus independent of TRPA1 channels. We further found that the TRPA1 antagonist HC030031 reduced glucose clearance in the glucose tolerance test very significantly in both *Trpa1^+/+^* and *Trpa1^−/−^* mice. HC030031 must therefore interfere with glucose tolerance independently of TRPA1 and this off-target effect suggests that results obtained with this compound should be interpreted with caution.

### TRPA1 as a mediator of diabetic neuropathy

Our results show that sustained elevations of endogenously formed MG by pharmacological inhibition of GLO-1 leads to the development of a painful diabetic neuropathy-like condition in mice. Activation of TRPA1 is essential for the observed sensory changes, since mice lacking TRPA1 appear unaffected by MG. TRPA1 and GLO1 are preferentially expressed in small-diameter, peptidergic DRG neurons [59], [60] and the GLO1 activity is reduced in DRG neurons from diabetic mice [47]. Although our results imply that increased formation of MG is sufficient to produce painful diabetic sensory neuropathy, it is likely that increased levels of reactive oxygen species and lipid peroxidation products also contribute to the development of sensory neuropathy in diabetes [5], [10], [11], [13].

Although diabetic neuropathy is a common cause of neuropathic pain, loss of sensation is the major clinical symptom. It is possible that a loss of peripheral sensory nerve fibers as a consequence of persistent activation of TRPA1 also contributes to the development of insensate features of diabetic sensory neuropathy [27]. We did not detect a loss of IENFs or a loss of sensation and it is possible that degeneration of epidermal fibers takes longer than the two weeks that we examined in this study. It is also possible that long-lasting inhibition of GLO-1 produced higher levels of MG than would be the case in *diabetes mellitus*. Hyperglycemia reduces the cellular concentration of reduced glutathione in DRG neurons, leaving the cells more vulnerable to oxidative insults [61]. Since glutathione is a necessary co-factor for GLO1, episodes of hyperglycemia or oxidant stress would also reduce the cellular capacity to convert MG to lactate [29]. Reactive oxygen species and electrophilic metabolites are potent endogenous TRPA1 agonists [4] and are important for the development of diabetic neuropathy [3], [62]. Oxidative stress and increased formation of MG thus converge on the nociceptive ion channel TRPA1 during hyperglycemia, thereby selectively stimulating nociceptive fibers.
