# Correction: Methylglyoxal Evokes Pain by Stimulating TRPA1

**DOI:** 10.1371/annotation/e707d50a-13b3-4cc3-b507-7d8360d8f048

**Published:** 2013-12-05

**Authors:** David A. Andersson, Clive Gentry, Emily Light, Nisha Vastani, Julie Vallortigara, Angelika Bierhaus, Thomas Fleming, Stuart Bevan

This paper has been republished on December 3 due to an incorrect author designation. Please download the article again to view the article with the corrected author byline.

